# Macrophages expressing uncoupling protein 1 increase in adipose tissue in response to cold in humans

**DOI:** 10.1038/s41598-021-03014-3

**Published:** 2021-12-08

**Authors:** Brian S. Finlin, Hasiyet Memetimin, Amy L. Confides, Beibei Zhu, Philip M. Westgate, Esther E. Dupont-Versteegden, Philip A. Kern

**Affiliations:** 1 The Department of Internal Medicine, Division of Endocrinology, CTW 521, Barnstable Brown Diabetes and Obesity Center, University of Kentucky, 900 S. Limestone St., Lexington, KY 40536 USA; 2 Department of Rehabilitation Sciences, College of Health Sciences and Center for Muscle Biology, University of Kentucky, Lexington, KY 40536 USA; 3 College of Public Health, University of Kentucky, Lexington, KY 40536 USA

**Keywords:** Obesity, Metabolic syndrome

## Abstract

Acute cold induces beige adipocyte protein marker expression in human subcutaneous white adipose tissue (SC WAT) from both the cold treated and contralateral leg, and the immune system regulates SC WAT beiging in mice. Cold treatment significantly increased the gene expression of the macrophage markers CD68 and 86 in SC WAT. Therefore, we comprehensively investigated the involvement of macrophages in SC WAT beiging in lean and obese humans by immunohistochemistry. Cold treatment significantly increased CD163/CD68 macrophages in SC WAT from the cold treated and contralateral legs of lean and obese subjects, and had similar effects on CD206/CD68 macrophages, whereas the effects on CD86/CD68 macrophages were inconsistent between lean and obese. However, linear regression analysis did not find significant relationships between the change in macrophage numbers and the change in UCP1 protein abundance. A high percentage of CD163 macrophages in SC WAT expressed UCP1, and these UCP1 expressing CD163 macrophages were significantly increased by cold treatment in SC WAT of lean subjects. In conclusion, our results suggest that CD163 macrophages are involved in some aspect of the tissue remodeling that occurs during SC WAT beiging in humans after cold treatment, but they are likely not direct mediators of the beiging process.

## Introduction

Subcutaneous white adipose tissue (SC WAT) of adult humans is a dynamic tissue that is capable of undergoing enormous expansion to store lipid during nutrient excess, or producing free fatty acids by lipolysis in response to demand. Chronic stimulation of the sympathetic nervous system by cold or specific agonism of βadrenergic receptors (βAR) receptors remodels SC WAT by inducing the formation of a unique type of adipocyte in white adipose tissue that is called a beige adipocyte^1^. Studies in mice have demonstrated that beige adipose has beneficial effects on metabolic homeostasis (recently reviewed^2^). Less is known about beige adipose in humans, but our recent work has demonstrated that human SC WAT increased the expression of beige adipose markers in response to cold or $\upbeta$3AR stimulation^3, 4^. In response to the β3AR agonist mirabegron, beiging occurred along with improvement of glucose metabolism and muscle fiber type switching to type I fibers in obese research participants^3^, and another study also found improved glucose metabolism after mirabegron treatment^5^.

Studies in mice have demonstrated a role for cells of the immune system in adipose beiging (recently reviewed^2, 6^). These studies have implicated macrophages, eosinophils, iNKT cells, and type 2 innate lymphoid cells in the process of SC WAT beiging with a number of distinct roles described for each cell type^6–12^. Immune cells interact with each other and beige adipocytes, secreting numerous proteins and small molecules that regulate multiple aspects of beige adipose tissue including sympathetic tone^6–16^. The role that macrophages play in SC WAT beiging in mice has been intensively studied, and these studies support the concept that alternatively activated, anti-inflammatory macrophages increase in SC WAT in response to sympathetic nervous system activation and are associated with beiging, whereas pro-inflammatory macrophages inhibit beiging^6, 7, 17–20^. Our recent studies on the role of immune cells in human SC WAT implicated mast cells in the seasonal regulation of UCP1 in humans^21^ and in beiging in response to acute cold^22^. Furthermore, we observed that a subtype of alternatively activated macrophages that express CD163 and are called M2c, increased in SC WAT after treatment with mirabegron, suggesting a potential role for these alternatively activated macrophages in beiging^3^.

In this study, we analyzed SC WAT of humans that had increased beige adipose marker expression in response to cold^4^. We detected changes in the gene expression of macrophage markers in obese subjects that were treated acutely with cold. We then comprehensively characterized SC WAT macrophages by immunohistochemistry and examined the relationship between macrophages and SC WAT beiging.

## Results

### Repeated cold exposure increases macrophage marker gene expression in SC WAT

We previously observed that repeated cold exposure (an ice pack applied to one leg for 30 min per day for 10 days) increased the expression of three beige adipose tissue protein markers (UCP1, TMEM26, and CIDEA) in SC WAT of both lean and obese research participants^4^. That study was designed to evaluate the direct effect of cold by analyzing SC WAT from the cold treated leg, and SC WAT from the contralateral leg was studied as well^4^. Interestingly, cold-induced beige adipose marker expression was equivalent in both legs, likely due to sympathetic nervous system activation^4^. Here, we performed multiplex analysis of gene expression in the SC WAT from the obese subjects of that study using the Nanostring nCounter system and a code set designed to measure immune cells markers and chemokines, extracellular matrix remodeling, angiogenesis, adipokines, and important metabolic genes and transcription factors^22^. Results of multiplex analysis of gene expression from the lean subjects were recently reported and identified an interesting role for mast cells in beiging^22^. In addition, we also note that results of gene expression of UCP1 and TMEM26 were also reported^4^. Genes that were significantly changed by acute cold exposure in SC WAT from the legs of obese subjects are shown in Tables 1 and 2. Analysis of these two tables suggested that cold affected macrophages since the gene expression of the pan macrophage marker CD68 and the pro-inflammatory macrophage marker CD86 was increased in SC WAT from both legs (Tables 1 and 2). There was also a trend (P = 0.06) for an increase in gene expression of the macrophage marker CD163 in SC WAT from both legs (Tables 1 and 2).

**Table 1:** Genes significantly regulated in SC WAT of the cold treated leg of obese research participants.

Gene^a^	Function	Pre counts	Pre SEM	Post counts	Post SEM	Fold change	P-value
FNDC5	Secreted factor (promotes beiging)	20	2	16	1	0.783	0.009
IL18	Cytokine	85	8	109	13	1.288	0.015
ADIPOR1	Adiponectin Receptor	2847	168	2232	163	0.784	0.020
CD68	Macrophage marker (pan)	1309	227	1646	302	1.257	0.024
TIMP2	Extracellular matrix remodeling	3475	251	3093	194	0.890	0.028
CCL18	Chemokine	703	238	1536	482	2.185	0.035
ANGPTL1	Angiogenesis regulator	345	23	282	38	0.818	0.036
F3	Secreted factor	709	58	551	61	0.777	0.042
EBF3	Brown fat marker	608	29	509	46	0.837	0.043
CD86	Macrophage marker (M1)	107	9	139	19	1.296	0.048
FGF2	Secreted factor	1289	113	1088	143	0.844	0.050
CD163	Macrophage Marker (M2)	1240	109	1639	239	1.322	0.06

^a^Gene expression was measured with a custom codeset^22^ using the Nanostring nCounter system. The expression level of the gene (nCounter counts) and the SEM are indicated. The fold-change in gene expression (post / pre) is also indicated. Data were analyzed by a paired, two-tailed Student’s t-test.

**Table 2:** Genes significantly regulated in SC WAT of the contralateral leg of obese research participants.

Gene^a^	Function	Pre counts	Pre SEM	Post counts	Post SEM	Fold change	P-value
EBF3	Brown fat marker	608	29	505	30	0.830	0.003
TEK	Angiogenesis	437	31	335	21	0.767	0.003
FOXO1	Transcription regulation	1037	40	858	41	0.827	0.006
ACACA	Fatty Acid metabolism	166	15	144	15	0.870	0.007
F3	Secreted factor	709	58	534	54	0.754	0.009
FGF2	Secreted factor	1289	113	1019	75	0.791	0.012
SIRT1	Transcription regulation	467	22	422	22	0.903	0.013
BCL2	Apoptosis	617	14	489	44	0.792	0.021
ANGPT4	Angiogenesis	14	2	11	1	0.784	0.023
LEP	Adipokine	4558	793	3900	594	0.856	0.023
HMOX1	Heme metabolism	942	84	3066	781	3.255	0.036
CCL18	Chemokine	703	238	1451	411	2.065	0.037
CD86	Macrophage marker (M1)	107	9	137	16	1.287	0.041
VEGFA	Angiogenesis	810	50	925	58	1.142	0.045
LOX	Extracellular matrix	1514	87	1792	117	1.183	0.047
CD68	Macrophage marker (pan)	1309	227	1712	306	1.308	0.048
CD163	Macrophage marker (M2)	1240	109	1575	139	1.270	0.061

^a^Gene expression was measured with a custom codeset^22^ using the Nanostring nCounter system. The expression level of the gene (nCounter counts) and the SEM are indicated. The fold-change in gene expression (post / pre) is also indicated. Data were analyzed by a paired, two-tailed Student’s t-test.

### Repeated cold exposure changes macrophage abundance in SC WAT

**Figure 1:**
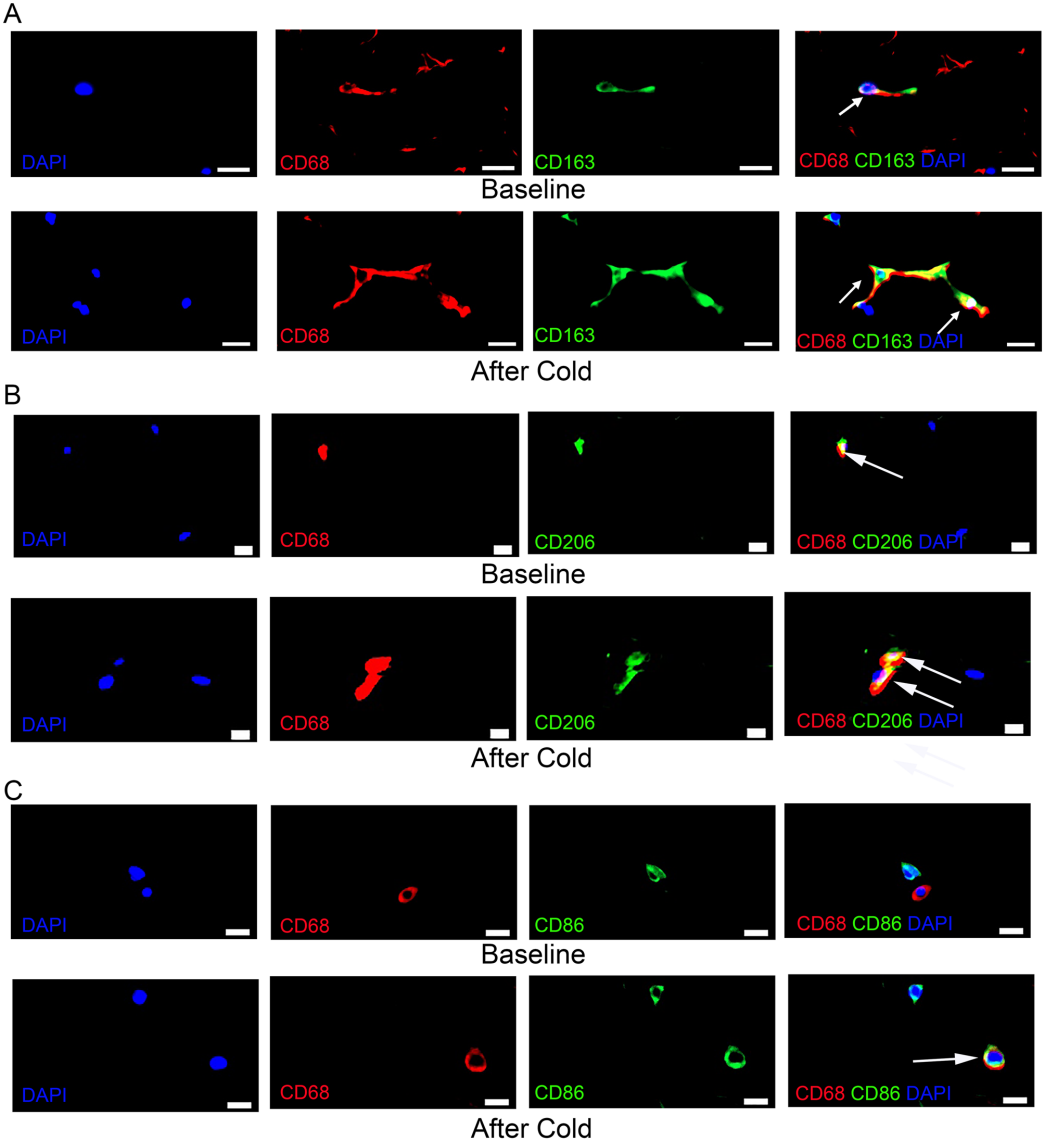
Representative images of macrophage immunohistochemistry. Human SC WAT was co-stained with antibodies against CD163 and CD68 (**A**), CD206 and CD68 (**B**), or CD86 and CD68 (**C**) before and after cold treatment as indicated. Fluorescence in each individual channel is presented followed by a merged image. Arrows indicate cells that co-stain with each specific macrophage antibody, CD68 (red), and that are DAPI positive (blue). Scale bars: 10 $\upmu$m.

**Figure 2:**
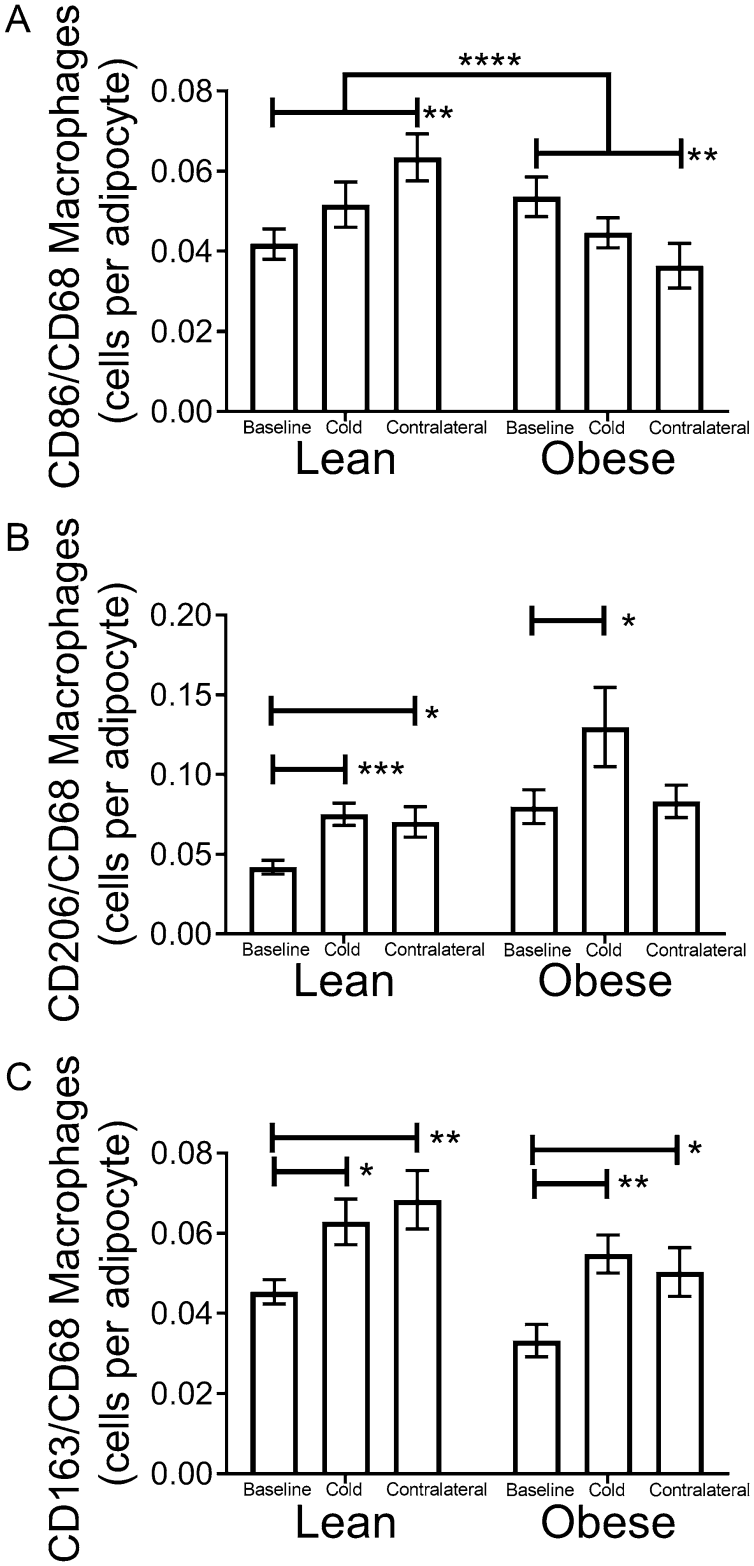
Quantification of inflammatory and anti-inflammatory macrophages in SC WAT of research participants in response to acute cold treatment. (**A**) to (**C**) Quantification of CD86/68, CD206/68, and CD163/68 positive macrophages in lean (n = 15–17) and obese (n = 8) research participants at baseline and in SC WAT from the cold and contralateral legs after 10 days of acute cold exposure. Data represent means ± SEM and were analyzed by RM MANOVA as described in research design and methods. *P < 0.05; **P < 0.01; ***P < 0.001; ****P < 0.0001 (lean n = 17; obese n = 8).

Since the analysis of gene expression suggested that macrophage abundance in SC WAT is changed by cold, we comprehensively quantified macrophages by immunohistochemistry using three common macrophage markers. These markers (CD86, CD163, and CD206) discriminate between pro-inflammatory (M1, CD86) and anti-inflammatory (M2, CD163 and CD206) macrophages. We note that the SC WAT was previously shown to have increased beige protein marker expression (TMEM26 and UCP1), and that UCP1 was shown to be expressed in additional structures besides unilocular adipocytes^4^. We used co-staining of the pan macrophage marker CD68 with CD86 to identify M1 macrophages, CD68 with C206 to identify M2 macrophages, and CD68 with CD163 to identify M2c macrophages. Representative staining of each type of macrophage is shown in Fig. 1. CD86/CD68 macrophages increased in the contralateral leg of lean subjects, but decreased in the contralateral leg of obese subjects (Fig. 2A; P < 0.01), and this difference in response between lean and obese subjects was highly significant (Fig. 2A; interaction P < 0.0001). Cold significantly increased CD206/CD68 macrophages in SC WAT of the cold treated leg of both lean and obese subjects (Fig. 2B; P < 0.001), but only in the contralateral leg of lean subjects (Fig. 2B; P < 0.05). Finally, cold significantly increased CD163/CD68 macrophages in SC WAT of the cold treated leg and of the contralateral leg of lean and obese subjects (Fig. 2C lean: P < 0.05 (cold), P < 0.01 (contralateral), obese: P < 0.01 (cold), P < 0.05 (contralateral)). Thus, cold treatment had the most consistent effect on increasing CD163/CD68 macrophages. Interestingly, our analysis of gene expression identified the chemokine CCL18 as being induced by approximately twofold in both legs (Tables 1 and 2). CCL18 is has been demonstrated to polarize macrophages to the M2 phenotype and to increase CD163 expression^23^, providing a possible additional mechanism besides recruitment for the consistent increase in CD163/CD68 macrophages. Notably, the enzyme heme oxygenase 1 (HMOX1), which is induced by CD163 signaling^24^, was highly increased by cold (Tables 1 and 2), consistent with the increase in CD163/CD68 macrophages.

**Figure 3:**
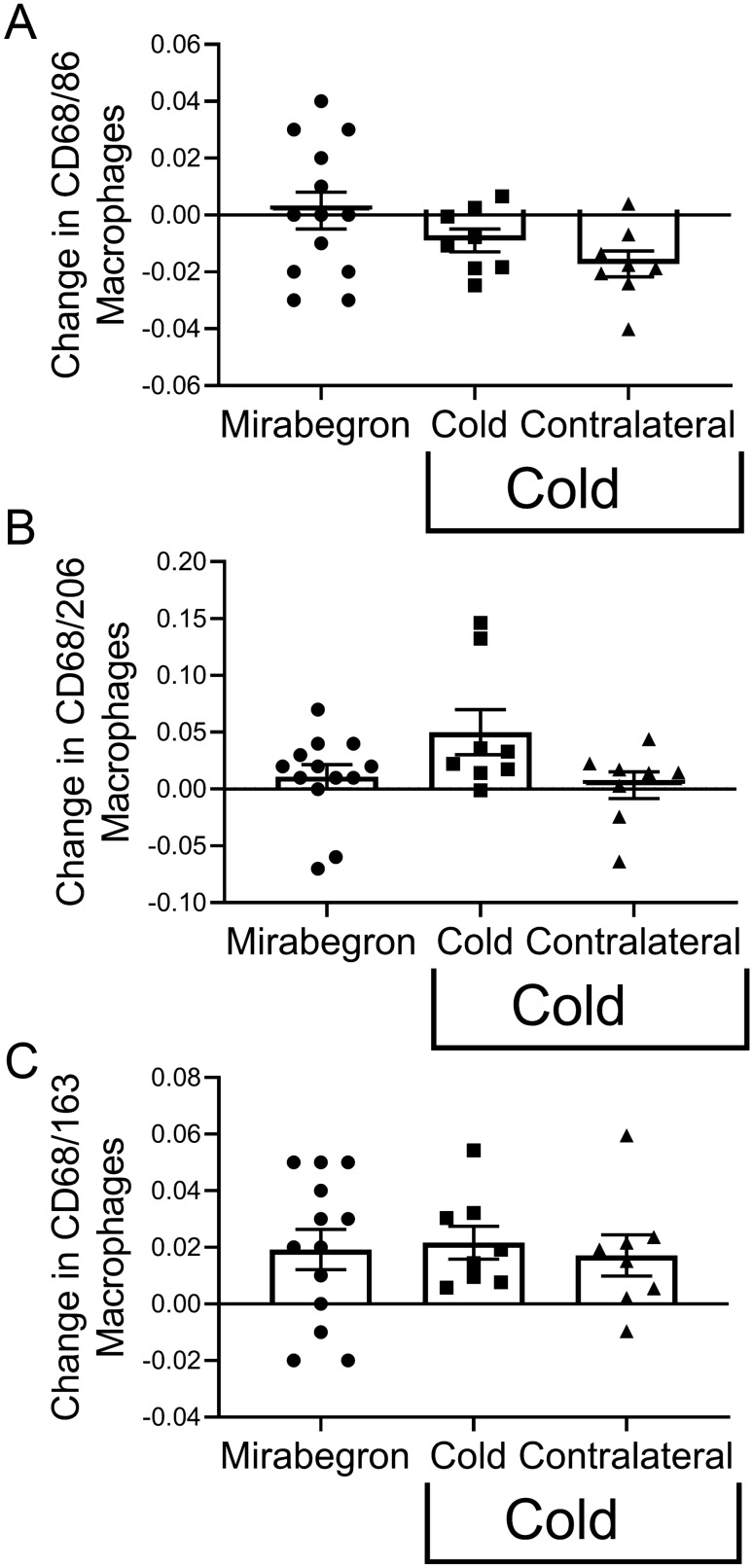
Analysis of the change in inflammatory and anti-inflammatory macrophages in SC WAT of obese research participants in response to mirabegron and acute cold treatment. The change (post–pre) in macrophages in SC WAT was calculated for obese subjects treated with cold or for subjects treated with mirabegron using previous published data^3^. (**A**) to (**C**) Analysis of the change in CD86/68, CD206/68, and CD163/68 positive macrophages in response to mirabegron (n = 13) or cold (n = 8). Data represent means ± SEM and were analyzed by ANOVA as described in research design and methods. *P < 0.05; **P < 0.01; ***P < 0.001; ****P < 0.0001 (lean n = 17; obese n = 8).

We previously reported that mirabegron treatment of obese subjects increased CD163/68 macrophages in SC WAT of obese subjects, but did not result in any change in CD86/68 or CD206/68 macrophage abundance in SC WAT^3^. This suggests that there are differences between the response to a $\upbeta$3AR agonist and cold. We therefore analyzed whether the change in macrophage recruitment by cold was different from the change caused by mirabegron. When we compared the responses of the subjects in these studies, we did not detect a significant difference in the change in CD86/68 or CD206/CD68 macrophages (Fig. 3A and B) in the relatively small number of subjects in each study. Similarly, the magnitude of the change in CD163/68 macrophages was similar among the treatments (Fig. 3C), consistent with the ability of each treatment to significantly increase this subtype of macrophage (Fig. 2C and^3^).

#### Cold increases CD163/UCP1 macrophages in SC WAT

**Figure 4:**
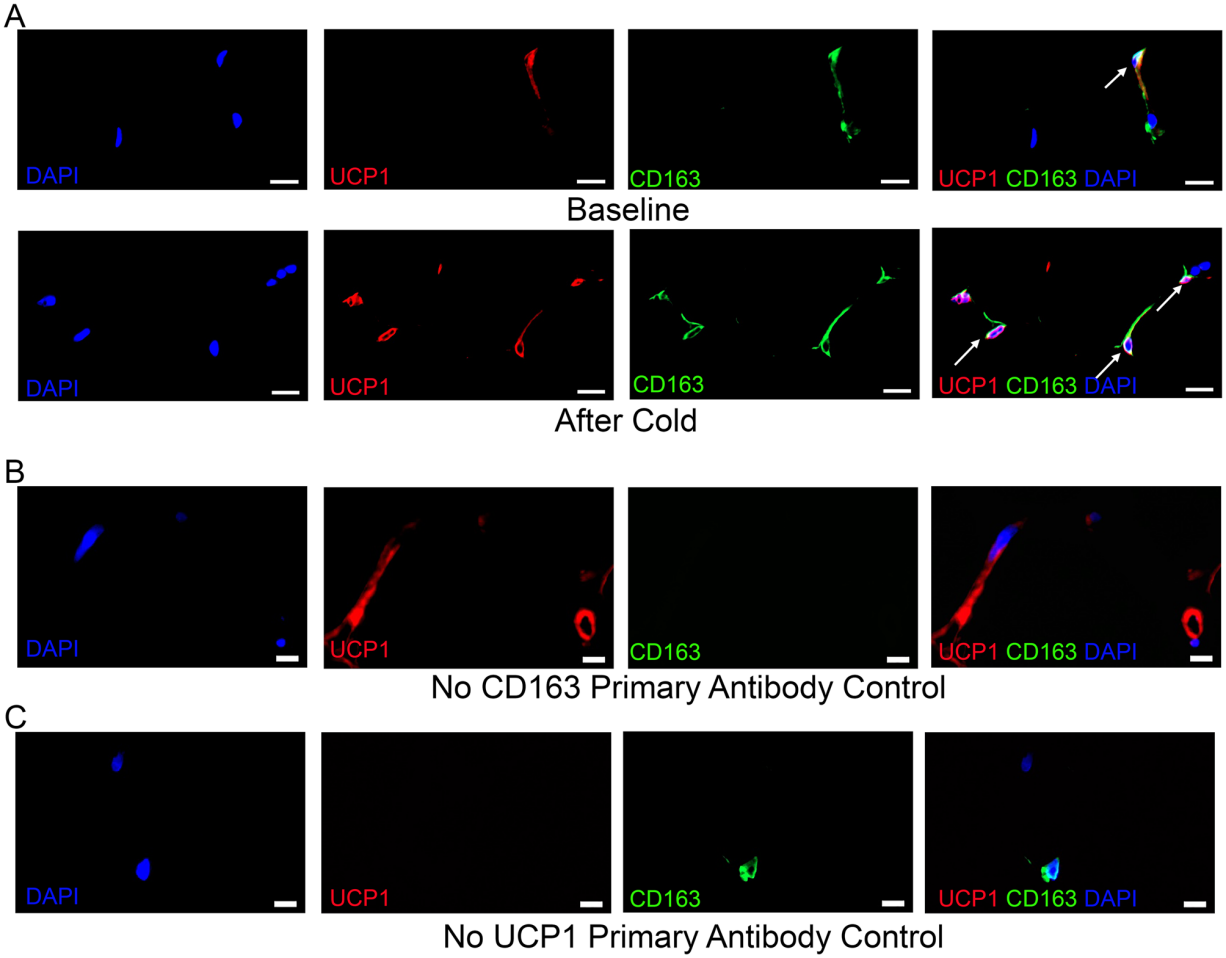
Representative images of UCP1 and CD163 co-staining. (**A**) Human SC WAT was co-stained with UCP1 and CD163 antibodies before and after cold treatment as indicated. Fluorescence in each individual channel is presented followed by a merged image. Arrows indicate cells that co-stain with UCP1 (red) and CD163 (green), and that are DAPI positive (blue). (**B**) and (**C**) No primary antibody controls for the co-staining are presented. Scale bars: 10 $\upmu$m.

**Figure 5:**
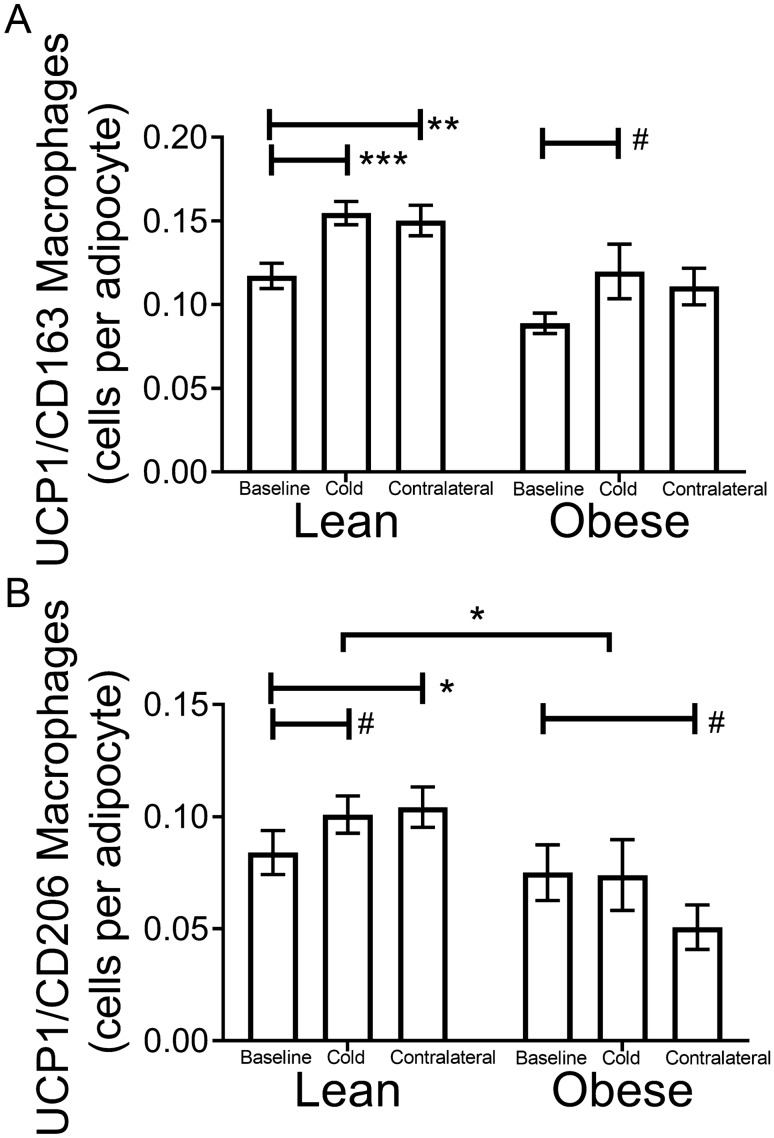
Quantification of UCP1 positive macrophages in SC WAT of research participants in response to acute cold treatment. (**A**) and (**B**) Quantification of CD163/UCP1 and CD206/UCP1positive macrophages in lean (n = 17) and obese (n = 8) research participants at baseline and in SC WAT from the cold and contralateral legs after 10 days of acute cold exposure. Data represent means ± SEM and were analyzed by RM MANOVA as described in research design and methods. *P < 0.05; **P < 0.01; ***P < 0.001; ^#^P < 0.1 (lean n = 17; obese n = 8).

**Figure 6:**
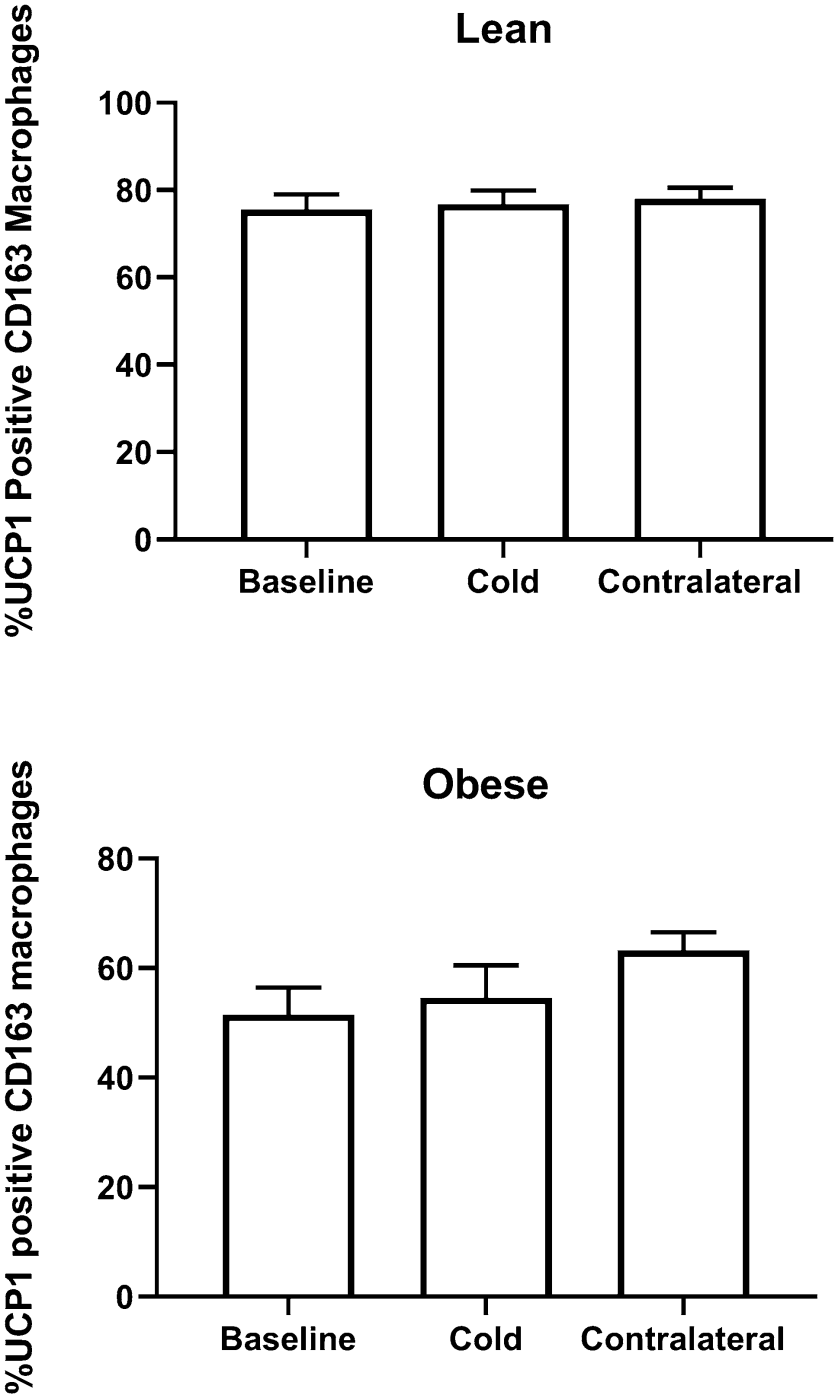
Quantification of the percentage of CD163 macrophages that express UCP1 in response to acute cold treatment. The percentage of CD163 macrophages that express UCP1 before and after cold is indicated. Data represent means ± SEM.

We recently observed that CD163 macrophages expressed UCP1 and that CD163/UCP1 positive cells increased in SC WAT following chronic treatment with the $\upbeta$3AR agonist mirabegron^3^. Representative images of UCP1-expressing CD163 positive cells are shown in Fig. 4. We quantified CD163/UCP1 cells in SC WAT and found that UCP1/CD163 positive cells increase in SC WAT from both the cold-treated and contralateral legs of lean subjects (Fig. 5A; cold lean: P < 0.01; contralateral lean: P < 0.001). In obese subjects, the increase of CD163/UCP1 macrophages in SC WAT from the cold treated leg was not statistically significant (P < 0.10). Approximately 75% of CD163 macrophages in lean subjects and 50% in obese subjects expressed UCP1, but this percentage did not significantly change after cold exposure (Fig. 6). Thus, the increase in CD163/UCP1 macrophages is due mostly to the increase in recruitment and/or phenotype switching to CD163 macrophages (Fig. 2C). Next, we examined UCP1/CD206 macrophages and found that they were only significantly increased in the contralateral leg (Fig. 5B; P < 0.05) with a trend for an increase in the cold treated leg of lean subjects. However, there was a trend for CD206/UCP1 macrophages being lower in the contralateral leg of obese subjects after cold (Fig. 5B; P < 0.1), and this different response between lean and obese in the contralateral legs was significant (P < 0.0001).

#### Macrophage recruitment does not predict the level of SC WAT beiging

We have recently characterized beiging in this cohort of lean and obese research participants in response to cold^4^. Some studies in mice have suggested that macrophages are direct mediators of beiging, perhaps as a source of catecholamine^7^, although this was recently disputed^12^. If macrophages are direct mediators of beiging, one would expect that subjects in which more macrophages were recruited to SC WAT would display a higher degree of beiging. We investigated this by performing a linear regression analysis of the change in UCP1 expression versus the change in macrophages in SC WAT from each leg. Overall there was little correlation (Table 3), suggesting that macrophages are recruited to adipose tissue for other purposes such as tissue remodeling, regulating local free fatty acid levels, or other putative roles suggested by rodent studies^6^. We also investigated whether the change in CD163 macrophages expressing UCP1 correlated with the change (increase) in UCP1 activity using bioenergetics data available from five of the lean subjects^4^, but did not find a significant correlation (P = 0.42). Finally, we performed regression analyses of the change in CD163 macrophages versus the change in the expression of several genes important for adipose function, such as adiponectin, PPAR$\upgamma$, and others, but did not observe any significant correlations (Table 4).

**Table 3:** Linear regression analysis of the change in macrophages and change in UCP1.

Macrophage type	Leg	r^2^	P-value^a^
**Lean research participants**			
CD206/CD68	Cold	0.026	0.56
CD206/CD68	Contralateral	0.330	0.02^b^
CD163/CD68	Cold	0.106	0.20
CD163/CD68	Contralateral	0.002	0.87
CD86/CD68	Cold	< 0.0001	0.99
CD86/CD68	Contralateral	0.076	0.28
**Obese research participants**			
CD206/CD68	Cold	0.033	0.67
CD206/CD68	Contralateral	0.117	0.41
CD163/CD68	Cold	0.204	0.26
CD163/CD68	Contralateral	0.002	0.91
CD86/CD68	Cold	0.066	0.54
CD86/CD68	Contralateral	0.028	0.69

^a^Linear regression analysis was performed on the change in macrophages (post–pre) versus the change in UCP1. The change in UCP1 was calculated from previously published data^4^. ^b^P < 0.05.

**Table 4:** Linear regression analysis of the change in CD163/CD68 macrophages and change gene expression.

Gene^a^	Leg	r^2^	P-value^b^
**Lean research participants**			
Adiponectin	Cold	0.006	0.81
Adiponectin	Contralateral	0.004	0.85
PPAR$\upgamma$1	Cold	0.10	0.31
PPAR$\upgamma$1	Contralateral	0.006	0.81
PPAR$\upgamma$2	Cold	0.13	0.26
PPAR$\upgamma$2	Contralateral	0.05	0.50
Fatty Acid Synthase	Cold	0.05	0.50
Fatty Acid Synthase	Contralateral	0.07	0.42
Leptin	Cold	0.17	0.18
Leptin	Contralateral	0.21	0.13
FABP4	Cold	0.24	0.10
FABP4	Contralateral	0.03	0.60
PGC1$\mathrm{\alpha }$	Cold	0.04	0.53
PGC1$\mathrm{ \alpha }$	Contralateral	0.05	0.50
**Obese research participants**			
Adiponectin	Cold	0.03	0.69
Adiponectin	Contralateral	0.13	0.38
PPAR$\upgamma$1	Cold	0.10	0.44
PPAR$\upgamma$1	Contralateral	0.30	0.16
PPAR$\upgamma$2	Cold	0.01	0.78
PPAR$\upgamma$2	Contralateral	0.05	0.59
Fatty Acid Synthase	Cold	0.003	0.90
Fatty Acid Synthase	Contralateral	0.07	0.51
Leptin	Cold	0.01	0.82
Leptin	Contralateral	0.01	0.78
FABP4	Cold	0.22	0.24
FABP4	Contralateral	0.29	0.17
PGC1$\mathrm{\alpha }$	Cold	0.23	0.23
PGC1$\mathrm{\alpha }$	Contralateral	0.15	0.34

^a^Linear regression analysis was performed on the change in CD163/CD68 macrophages (post–pre) versus the change in the expression of the indicated gene.

## Discussion

This study demonstrated that anti-inflammatory (M2) macrophages, especially those expressing CD163, are recruited to SC WAT in both lean and obese subjects in response to cold. This observation is consistent with our recent finding that CD163 macrophages are increased in SC WAT by mirabegron treatment^3^, suggesting an important role for CD163 macrophages in SC WAT beiging induced by a specific $\upbeta$3AR agonist. A number of roles for macrophages has been proposed in tissue remodeling that occurs in response to cold, including macrophages being a source of catecholamine and direct mediators of beiging^7^, although this was recently disputed^12^. We did not observe significant correlation of increased CD163 macrophages with the increase in UCP1 protein expression, which was previously documented^4^, suggesting that macrophages are not direct mediators of beiging. This finding is similar to a recent study in mice^17^, suggesting that M2 macrophages are involved in other aspects of the changes that occur in SC WAT response to sympathetic nervous system activation such as tissue remodeling.

The role of macrophages in adipose tissue under different physiological settings such as obesity has been widely studied^25, 26^. Macrophages are key mediators of low grade adipose tissue inflammation in obesity, promoting insulin resistance^26^. Recent work indicates that adipose tissue inflammation also inhibits the formation of beige adipose and that M2 macrophages have numerous roles in beige adipose tissue (reviewed in^6^). The recruitment of anti-inflammatory macrophages, in particular CD163/CD68 positive macrophages, by cold could thus promote beiging by reducing SC WAT inflammation or additional mechanisms such as beige adipogenesis^27, 28^. There have been a limited number of studies on the effect of $\upbeta$AR agonism on SC WAT macrophages in humans^3^. The current study illustrates that cold has complicated effects on SC WAT macrophage abundance that depend on whether the subject is lean or obese and the type of macrophage being studied. The decrease of CD86/CD68 macrophages and increase of anti-inflammatory macrophages is predicted to reduce SC WAT inflammation, and determining whether acute cold improves adipose tissue function and/or metabolic homeostasis is an important goal for future studies.

CD163 macrophages were consistently increased in SC WAT by cold in parallel with increased beiging herein and in our previous study with the $\upbeta$3AR agonist mirabegron^3^, suggesting an important role for this type of macrophage in adipose beiging. One possible clue to the role of CD163 macrophages is the significant number co-expressing UCP1. CD163 macrophages may themselves be thermogenic, but the relatively low abundance of these macrophages does not make it likely that they make a substantial contribution to thermogenesis. Macrophages have been shown to increase in SC WAT in order to regulate local free fatty acid levels in response to lipolytic stimuli by taking up lipid^29^; increased uncoupled respiration would allow macrophages to oxidize some of the lipid to facilitate this process. The role of UCP1 in CD163 macrophages will thus require further investigation. Another possible role for the increase in CD163 macrophages role is suggested by CD163 itself. CD163 is the receptor for hemoglobin, and CD163 macrophages are known to be involved in iron homeostasis^30^, which is important in adipose beiging^31^. Interestingly, HMOX is regulated by CD163 engagement and was highly induced in SC WAT by cold (Tables 1 and 2), suggesting a possible regulatory role in iron metabolism during SC WAT beiging.

In conclusion, the results of this study suggest that cold significantly increases the abundance of CD163 macrophages in SC WAT. This increase is accompanied by increased beiging, and is consistent with our recent observation that the $\upbeta$3AR agonist mirabegron increased CD163 macrophage abundance in SC WAT and SC WAT beiging^3^. Identifying the role that CD163 expressing macrophages play in response to $\upbeta$AR agonism will require future investigation.

## Research design and methods

### Human subjects and study design

The baseline characteristics and additional details about the research participants have been described elsewhere^4^. The study was performed in subjects recruited in summer (between June 15 and September 1). SC WAT biopsies were obtained at baseline and after a cold pack was applied to the thigh 30 min per day for 10 days. Both the cold treated leg and the contralateral leg were biopsied after treatment. The change in UCP1 protein expression was calculated using previously published data^4^. All subjects gave informed consent, and the protocols were approved by the Institutional Review Board at the University of Kentucky. All experiments were performed in accordance with relevant guidelines and regulations. The Clinicaltrials.gov registration identifiers are NCT02596776 (date of registration: 11/04/2015) and NCT02919176 (date of registration 9/29/2016).

### mRNA quantification

We used the Nanostring ncounter multiplex system to measure the expression of 130 genes and six housekeeping genes in purified RNA from SC WAT of subjects with obesity in which we demonstrated beiging in response to cold^4^. Briefly, RNA was purified using RNAeasy Lipid Tissue minikits (Qiagen, Valencia, CA) and analyzed using an Agilent 2100 bioanalyzer. Gene expression was normalized to the geometric mean of the six housekeeping genes according to the manufacturer’s instructions. The genes in the code set are described in references^22, 32^.

### Immunohistochemistry

Immunohistochemistry on SC WAT sections was performed as described previously^22^. Briefly, paraffin embedded adipose sections were deparaffinized followed by antigen retrieval in 10 mM sodium citrate pH 6.5 at 92 °C. For CD206/CD68 macrophage staining, sections were blocked with 3% hydrogen peroxide, the streptavidin/biotin blocking kit (Vector #SP-2002), and then 2.5% normal horse serum. Phosphate buffered saline (PBS) was used as the antibody diluent. 2.5% normal goat serum was used as a blocking agent between primary antibodies. For all other co-staining, 2.5% horse serum was used for blocking, 1% horse serum was used as the antibody diluent for the first primary antibody, 5% horse serum was used for blocking between the first and subsequent primary antibodies, and 1% horse serum was used as the antibody diluent for the subsequent primary antibody. Secondary antibodies used were biotinylated donkey anti-rabbit (Jackson ImmunoResearch, 711–065-152), goat anti-mouse biotinylated (Jackson ImmunoResearch, 115–065-205), donkey anti-goat biotinylated (Jackson ImmunoResearch, 705–065-147), or goat anti-rabbit biotinylated (Jackson ImmunoResearch, 111–065-045). Slides were incubated with strepavidin-HRP (#S911, Life Technologies, Carlsbad, CA), and then AlexaFluor 488 or 594 tyramide reagent (#B40957, Invitrogen, Carlsbad, CA) to visualize antibody binding. Sections were mounted in Vectashield with DAPI (H1200; Vector Laboratories). Macrophages were identified in SC WAT as DAPI positive cells that co-stained with CD68, which was used as a pan macrophage marker, and the indicated macrophage marker. The primary antibody combinations used for co-staining were as follows: CD206/CD68 (R&D systems AF2534; Dako #M0814), CD86/CD68 (Abcam, ab53004; Abcam, ab955), CD163/CD68 (Abcam, ab182422, Abcam, ab955), CD163/UCP1 (Hycult, HM2157, ECM Biosciences, J2648), CD206/UCP1 (R&D systems, AF2534, ECM Biosciences, J2648). Slides were analyzed with a Zeiss AxioImager MI upright fluorescent microscope (Zeiss, Gottingen, Germany) and Zen software (Zeiss).

### Statistics

Paired student’s two-tailed t-tests on gene expression, one-way ANOVAs, and linear regression analyses were performed in Graphpad prism. Repeated measures multivariate analysis of variance (RM MANOVA) was performed as described^4^ to analyze macrophage recruitment using SAS version 9.4. Immunohistochemistry was performed in a blinded manner.

## Data Availability

The datasets generated during and/or analyzed during the current study are available from the corresponding author on reasonable request.
